# Detection of Anatoxin-a and Three Analogs in *Anabaena* spp. Cultures: New Fluorescence Polarization Assay and Toxin Profile by LC-MS/MS

**DOI:** 10.3390/toxins6020402

**Published:** 2014-01-24

**Authors:** Jon A. Sanchez, Paz Otero, Amparo Alfonso, Vitor Ramos, Vitor Vasconcelos, Romulo Aráoz, Jordi Molgó, Mercedes R. Vieytes, Luis M. Botana

**Affiliations:** 1 Department of Pharmacology, Veterinary School, University of Santiago de Compostela, Lugo 27002, Spain; E-Mails: jonandoni.sanchez@rai.usc.es (J.A.S.); mariapaz.otero@rai.usc.es (P.O.); amparo.alfonso@usc.es (A.A.); 2 Department of Physiology, Veterinary School, University of Santiago de Compostela, Lugo 27002, Spain; E-Mail: mmercedes.rodriguez@usc.es; 3 Department of Biology, Faculty of Sciences, University of Porto, Rua do Campo Alegre, Porto 4619-007, Portugal; E-Mails: vtr.rms@gmail.com (V.R.); vmvascon@fc.up.pt (V.V.); 4 Center of Marine and Environmental Research—CIMAR/CIIMAR, University of Porto, Rua dos Bragas, 289, Porto 4050-123, Portugal; 5 CNRS, Institut de Neurobiologie Alfred Fessard—FRC2118, Laboratoire de Neurobiologie et Développement—UPR3294, 1 Avenue de la Terrasse, Gif sur Yvette Cedex 91198, France; E-Mails: araoz@inaf.cnrs-gif.fr (R.A.); Jordi.Molgo@inaf.cnrs-gif.fr (J.M.)

**Keywords:** anatoxin-a, nicotinic acetylcholine receptor, fluorescence polarization, liquid chromatography-mass spectrometry, *Anabaena* spp*.*

## Abstract

Anatoxin-a (ATX) is a potent neurotoxin produced by several species of *Anabaena* spp*.* Cyanobacteria blooms around the world have been increasing in recent years; therefore, it is urgent to develop sensitive techniques that unequivocally confirm the presence of these toxins in fresh water and cyanobacterial samples. In addition, the identification of different ATX analogues is essential to later determine its toxicity. In this paper we designed a fluorescent polarization (FP) method to detect ATXs in water samples. A nicotinic acetylcholine receptor (nAChR) labeled with a fluorescein derivative was used to develop this assay. Data showed a direct relationship between the amount of toxin in a sample and the changes in the polarization degree of the emitted light by the labeled nAChR, indicating an interaction between the two molecules. This method was used to measure the amount of ATX in three *Anabaena* spp. cultures. Results indicate that it is a good method to show ATXs presence in algal samples. In order to check the toxin profile of *Anabaena* cultures a LC-MS/MS method was also developed. Within this new method, ATX-a, retention time (RT) 5 min, and three other molecules with a mass *m*/*z* 180.1 eluting at 4.14 min, 5.90 min and 7.14 min with MS/MS spectra characteristic of ATX toxin group not previously identified were detected in the *Anabaena* spp*.* cultures. These ATX analogues may have an important role in the toxicity of the sample.

## 1. Introduction

**Figure 1:**
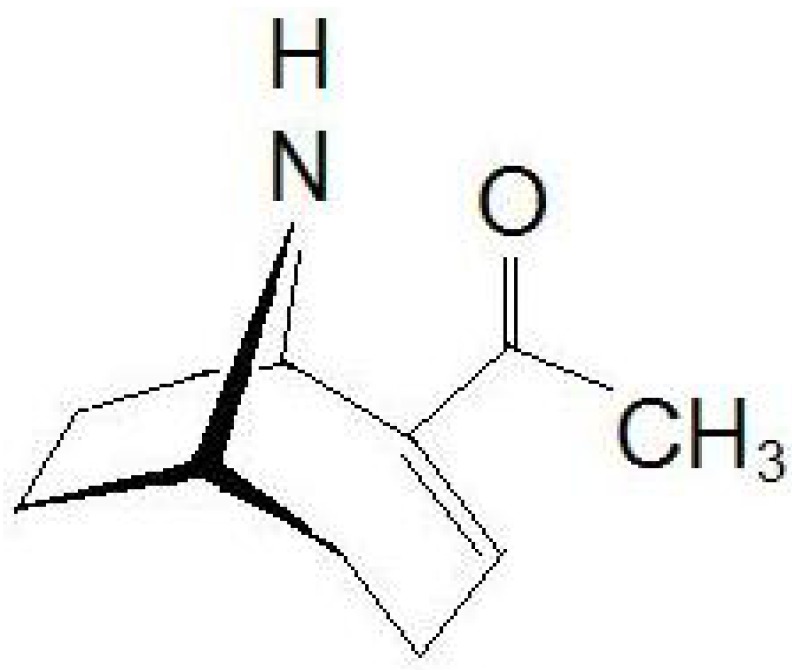
Chemical structure of anatoxin-a (ATX).

Freshwater toxins can be divided into three groups including microcystins and nodularin that produces hepatotoxic effects, anatoxin-a (ATX), homoanatoxin-a (HATX) and saxitoxins with neurotoxic effects and cylindrospermopsin that inhibits protein synthesis and induce oxidative stress [1]. ATX is a potent neurotoxic alkaloid produced by the cyanobacterium *Anabaena flos-aquae*. Nevertheless other species like *Anabaena planctonica*, *Oscillatoria acuminata*, *Aphanizomenon gracile* and *Cylindrospermun stagnale* were described as ATX producers [2,3]. Chemically, ATX (Figure 1) has a semi-rigid bicyclic secondary amine structure, 2-acetyl-9-azabicyclo[4:2:1]non-2-ene (C_10_H_15_NO). Both ATX and HATX produce rapid death of animals by respiratory paralysis and acute asphyxia, since these alkaloids are potent agonists of nicotinic acetylcholine receptors (nAChR) [4,5,6,7]. In mouse, the LD_50_ after intraperitoneal administration is 375 µg kg^−1^ body weight, while LD_50_ after oral consumption is higher than 5000 µg kg^−1^ body weight [8,9]. Besides the acute mouse death, under a high dose, ATX can act as a tumor promoter, and may cause cytotoxic or teratogenic effects after continuous low dose intake [9].

Cyanobacteria-producing cyanotoxins have a global distribution and most of them are able to bloom in aquatic environments, posing human health and environmental risks. The largest part of the outbreaks associated with this increasingly occurring phenomenon is being observed in water bodies of North America, Australia and Europe [8]. The appearance and duration cyanobacterial bloom seasons mainly depends on the climatic and environmental conditions of the region, such as the air temperature, concentration of nutrients, pH, salinity, sunlight irradiance, among others [10]. Connected with the increase in the number of cyanobacterial blooms worldwide is the increase of observations of animal intoxications due to ATX, in the last years. ATXs were linked to dog fatalities in California (USA), Ontario (Canada) and recently in The Netherlands [11,12]. Therefore, as stated before, massive proliferation of cyanobacteria constitutes a potential danger for humans, hence the potential toxin production needs to be monitored to prevent fatal episodes after water ingestion. Moreover, the first detection of ATX in human dietary supplements containing cyanobacteria gave rise to the increased risk of intoxications derived from possible intake of toxins through contaminated food [13]. Apart from toxins, cyanobacterial cells might produce many other metabolites, including toxic degradation products. For instance, epoxyanatoxin-a (EpoxyATX) and dihydroanatoxin-a (H_2_ATX) are products derived from ATX [14]. Since these metabolites had been detected in contaminated food supplements, they should be controlled in order to protect consumers [15]. In this sense, the risks of poisoning from contaminated water and/or food should be better monitored with sensitive techniques that confirm the presence or absence of these toxins. Most of the current methods for the detection of cyanobacterial ATXs require sophisticated methodology designs and instruments [16,17,18]. There is also a lack of reference toxins and standardized assays for the survey of this class of toxins. In the present study, a new and simple fluorescence polarization (FP) method that detects and quantifies ATX in natural samples of *Anabaena* spp*.* by binding the nAChRs from *Torpedo marmorata* membrane is described. In addition, a sensitive LC-MS/MS method for ATX detection is developed in order to identify and separate different toxin analogs. The total amount of toxins are quantified and compared by both methods.

## 2. Results and Discussion

**Figure 2:**
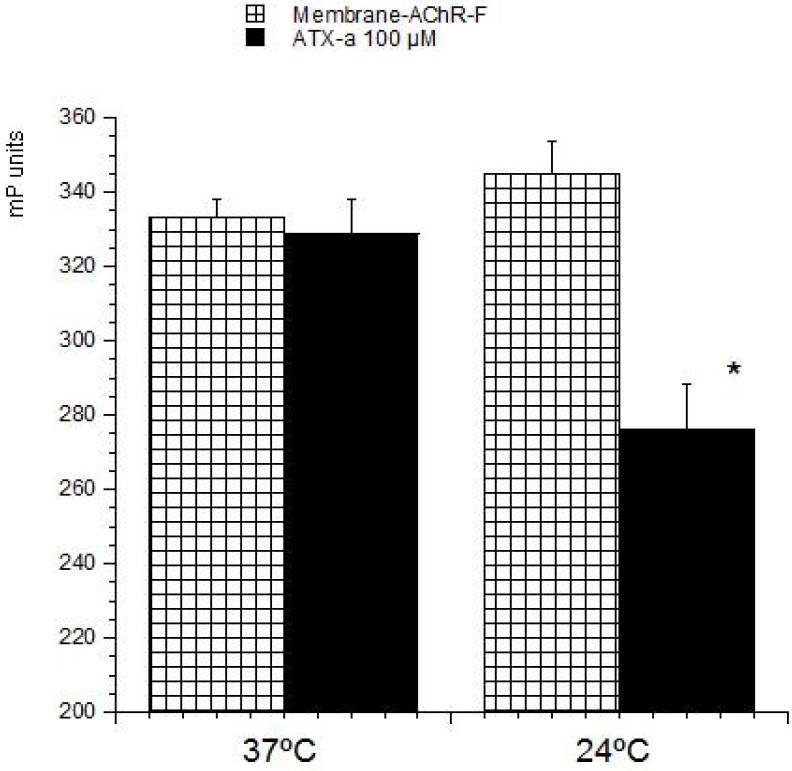
Effect of ATX-a on fluorescence polarization (FP) units (mP) of membrane-nAChR-F conjugate. 100 µM of ATX-a was added to a constant amount of membrane-nAChR-F conjugate. FP was measured after 60 min incubation at 37 °C and 24 °C. Data are means ± SEM of three experiments. (*) Significant differences with respect to membrane-nAChR-F conjugate.

**Figure 3:**
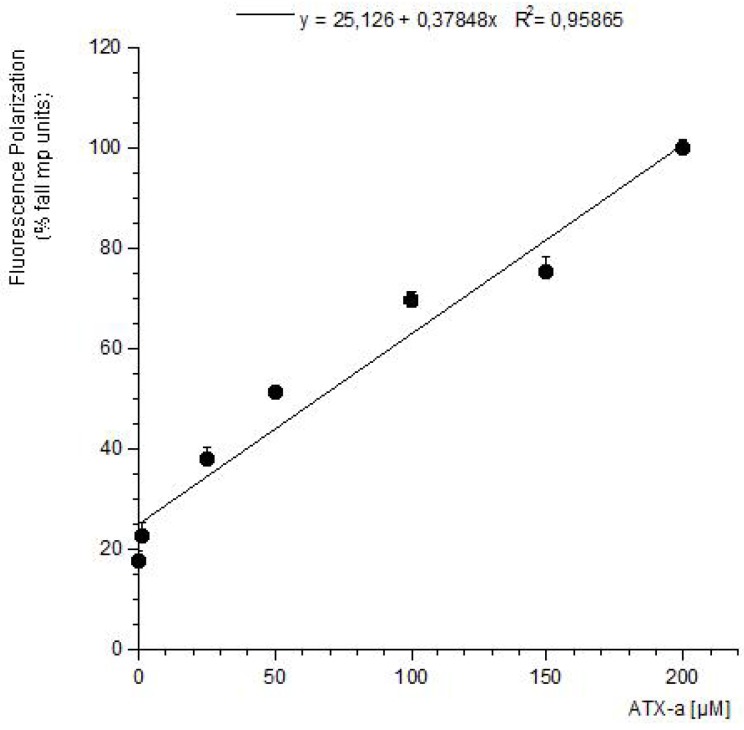
Effect of ATX-a on FP units (mP) of membrane-nAChR-F conjugate. Different concentrations of ATX-a were added to a constant amount of membrane-nAChR-F conjugate. FP was measured after 60 min incubation. Data are means ± SEM of three experiments.

**Figure 4:**
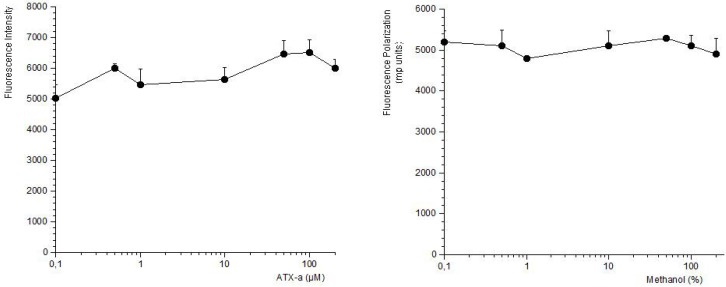
Effect of ATX-a (**left**) or methanol (**right**) on fluorescence intensity of membrane-nAChR-F conjugate. Different concentrations of ATX-a/methanol were added to a constant amount of membrane-nAChR-F conjugate. Fluorescence intensity was measured after 60 min incubation. Data are means ± SEM of three experiments.

FP is a suitable technique to study interactions between two molecules and therefore often used to develop biotoxins detection methods [19,20,21,22,23]. These methods are based on the ability of these compounds to bind with its intracellular target. Therefore this strategy was used to develop a detection method for ATX based on its association with the nAChR. The toxin-receptor interactions were measured as the FP variation (mP units) of a membrane-nAChR-F conjugate in the presence of different concentrations of ATX. First the membrane-nAChR-F conjugate was incubated with 100 µM of ATX-a. Different FP measures were done after 15, 30, 60 and 90 min of incubation at 37 °C and room temperature (24 °C) and continuous shaking, 300 rpm. Within these conditions, as Figure 2 shows, no changes in FP units of conjugate in the presence of ATX were observed after 60 min, at 37 °C. However, when the incubation was done at 24 °C a decrease of 70 mP units from 345 (no toxin) to 276 mP units (100 µM ATX) was observed. This fall indicates an interaction between the toxin and the conjugate. The FP variation was then studied by using different ATX-a concentrations, from 0.1 to 200 µM, and a constant amount of membrane-nAChR-F conjugate. As Figure 3 shows, an increase in the mP units fall was observed when the toxin concentration increased. These data fitted to a straight line that can be used to calculate the amount of ATX in a problem sample. The information provided by FP (the decrease) was lost when total fluorescence intensity (no polarized fluorescence) was tested (Figure 4-left). When different concentrations of ATX were added the fluorescence intensity of membrane-nAChR-F conjugate remained in a constant average value, with slight variations that cannot be related with the concentration of drug used. On the other hand when the solvent effect in fluorescence intensity was studied, Figure 4-right, no modifications were observed even with the highest concentration of methanol used (3%). All these results point to the measure of mP units of the membrane-nAChR-F conjugate as a good tool to check the ATX association and as a useful method to detect the toxin in a sample. In this way the limit of detection (LOD) and limit of quantification (LOQ) for ATX calculated under these conditions was 33.3 nM and 100 nM, respectively. These limits are higher than from other techniques [16]. However the FP method is simple, cost-effective, with a high degree of repeatability and fast. 

**Figure 5:**
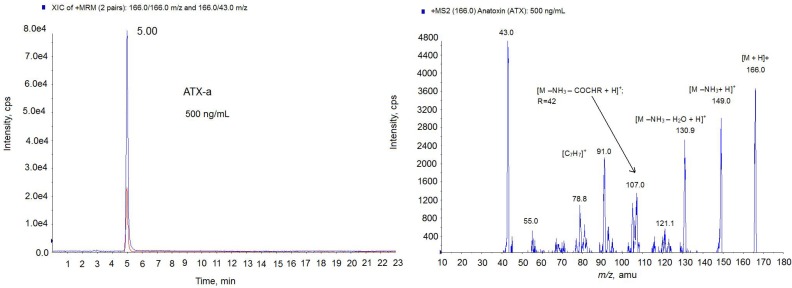
Multiple Reaction Monitoring (MRM) chromatogram in positive mode (**left**) and MS2 spectrum (**right**) of ATX standard (500 ng/mL) on the triple quadrupole mass spectrometer (QTRAP) instrument. Precursor ion: 166.0 *m*/*z*; Collision Energy (CE) 30; cps: counts per second.

**Figure 6:**
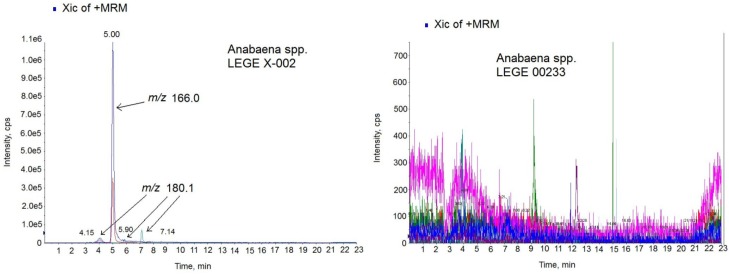
Multiple Reaction Monitoring (MRM) chromatogram in positive mode of the *Anabaena* spp. LEGE X-002 extract (**left**) and *Anabaena* sp. LEGE 00233 extract (**right**) on the triple quadrupole mass spectrometer (QTRAP) instrument. Transitions monitored in the MS method: anatoxin-a (ATX) (*m*/*z* 166 > 166, *m*/*z* 166 > 43), homoanatoxin-a (HATX) (*m*/*z* 180.1 > 163.1, *m*/*z* 180.1 > 145.1), dihidroanatoxin (H_2_ATX) (*m*/*z* 168.0 > 133.0, 168.0 > 150.0), dihidrohomoanatoxin (H_2_HATX) (*m*/*z* 182.0 > 147.0, 182.0 > 164.1), epoxyanatoxin-a (EpoxyATX-a) (*m*/*z* 182.0 >164.1, 182.0>138.1) and epoxyhomoanatoxin-a (EpoxyhomoATX-a) (*m*/*z* 196.0 > 178.2, 196.0 > 138.1).

Then, the FP method developed was tested with culture samples from three different cyanobacterial isolates, *Anabaena* sp. LEGE X-002, (ATX+, *i.e.*, ATX-a producer), *Anabaena* sp. LEGE 00233, (ATX-) and *Anabaena* sp. LEGE 00234, (ATX-). In these conditions, the amount of toxin detected in the ATX+ culture was 203.8 µg of ATX/mg biomass, while no toxin presence was detected in the ATX-cultures. The presence of ATX in *Anabaena* spp*.* cultures was also checked by LC-MS/MS technique. First, the LC-MS/MS method was developed and optimized with the ATX-a standard. As the Figure 5 chromatogram shows, 5 min was the retention time for the ATX-a standard (500 ng/mL). The spectrum obtained shows the typical losses and structures of the protonated ATX [24]. That is, the ion *m*/*z* 149 which corresponds to [M − NH_3_ + H]^+^ and the ion *m*/*z* 130.9 which indicates 1 loss of water from this molecule [M − NH_3_ − H_2_O + H]^+^. Since the highest ion was achieved with the mass *m*/*z* 43, the transition *m*/*z* 163 > 43 was chosen for the identification of ATX. After MS/MS parameters optimization, 1.5 ng ATX/mL (5.33 nM) as LOD and 5 ng ATX/mL (17.77 nM) as LOQ were obtained. In this method, an acid mobile phase composed by water and acetonitrile with 0.05% formic acid was used in gradient conditions with 23 min injections. Once the LC-MS/MS method was optimized, the next step was to check for the presence of ATXs in the extract of *Anabaena* spp. LEGE X-002. The sample was analyzed in positive MRM mode searching for the transitions of the ATX and the most common ATX analogues described in cultures and water food samples: HATX, H_2_ATX, H_2_HATX, EpoxyATX-a and EpoxyhomoATX-a [11,14,25,26]. Since ATX-a was the only available standard, the MS/MS settings used for the ATX analogs were those optimized for the ATX standard. The chromatogram of the sample in positive MRM mode showed 4 peaks with different intensity and mass (Figure 6-left). One prominent peak with a mass of *m*/*z* 166.0 eluting in the same time like the standard (5.00 min) and 3 small peaks with a mass of *m*/*z* 180.1 which eluted close to the high intensity peak at 4.15 min, 5.90 min and 7.14 min. The big peak with the same retention time as the standard is ATX-a and it was quantified by comparing the analytical standard peak with the peak area detected in the sample. The amount quantified was 13.32 µg ATX/mg biomass. The negative (ATX-) *Anabaena* spp. cultures were also analyzed for the same LC-MS/MS method and no peaks were detected. Figure 6-right shows the chromatogram of the negative culture LEGE 00233.

**Figure 7:**
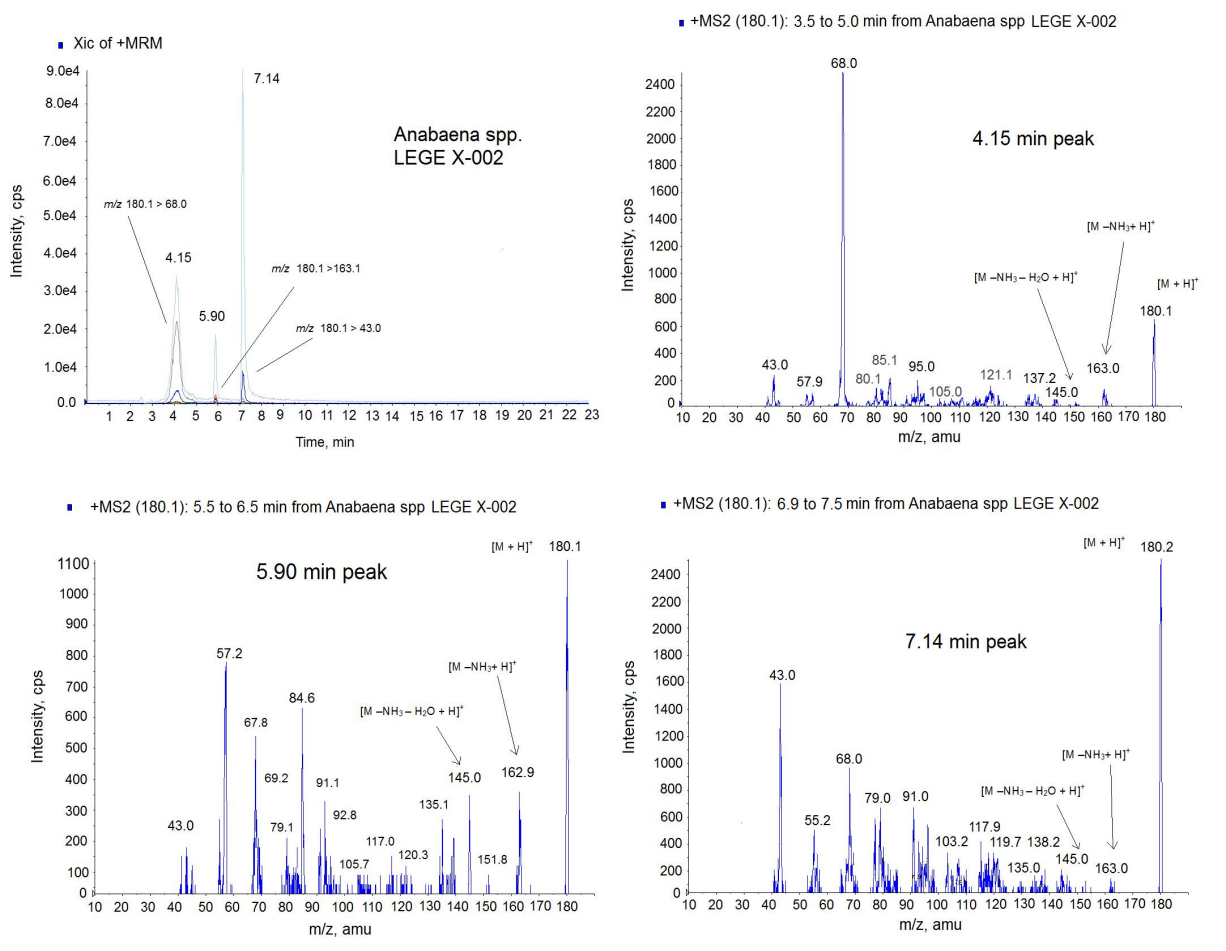
Positive multiple reaction monitoring (MRM) mode chromatogram and MS spectra of the 4.15 min peak, 5.90 min peak and 7.14 min peak from *Anabaena* spp*.* LEGE x-002 extract on the triple quadrupole mass spectrometer (QTRAP) instrument. Transitions monitored in the MRM method: *m*/*z* 180.1 > 180.1, *m*/*z* 180.1 > 163; *m*/*z* 180.1 > 145.0, *m*/*z* 180.1 > 68, *m*/*z* 180.1 > 43. The precursor ion in the MS2 mode: *m*/*z* 180.1. Collision Energy (CE): 30.

Due to the different intensity found among ATX (*m*/*z* 166.0) and the small molecules of mass *m*/*z* 180.1, the identification of the lastones was done separately. Since *m*/*z* 180.1 is the mass of the analog HATX and it was described in *Anabaena* sp. [24], we suspected that almost one of these peaks could be this toxin, therefore only the transitions common for HATX were monitored in this new approach, Figure 7. The MRM was done in positive mode monitoring the following transitions: *m*/*z* 180.1 > 180.1, *m*/*z* 180.1 > 163; *m*/*z* 180.1 > 145.0, *m*/*z* 180.1 > 68, *m*/*z* 180.1 > 43. As Figure 7 shows, 3 peaks contain all these transitions but in different ratio indicating that they are different compounds. For example the high intense transition for the 7.14 peak is *m*/*z* 180 > 43 and not for the two other peaks. This ratio between ions is shown in the MS spectra of the Figure 7. Although ATX analogue standards are not available, several issues suggest that they belong to the ATX toxin group. First, these peaks have the same mass as the HATX molecule, *m*/*z* 180 [17]. Second, the ionization is similar to the ATX-a standard, this is, 3 peaks has the mass *m*/*z* 163 with corresponds to [M − NH_3_ + H]^+^ and *m*/*z* 145 which indicates 1 loss of water from this molecule [M − NH_3_ − H_2_O + H]^+^. Third, several ions of the MS (*m*/*z* 68, *m*/*z* 91, *m*/*z* 135) are those shown for HATX spectrum [24]. And finally, three molecules have the ion *m*/*z* 43 which is typical of the ATX [27]. Therefore, these peaks appearing in the sample are ATX analogues. From the retention time, probably one of these two last peaks is HATX, because they elute after ATX [25], and the others two peaks, which are not described in the literature, correspond to the ATX family. Therefore, the different amount obtained by the FP assay and the LC-MS/MS technology are probably due to the presence of these new analogues that should be optimized and quantified. It is important to note that the response of each ATX in the MS detector is different despite the fact that the analysis conditions are the same and ATX-a cannot be used as universal standard for all derivatives [14]. As it is showed in Figure 5, with CE = 30 the maximum intensity for ATX standard is for the ion 43 *m*/*z* (4800 cps). This mass is followed in height by the ion *m*/*z* 166 due to [M + H]^+^ with a height of 3600 cps. However, in the spectrum of the analogues (Figure 7) for the same conditions, the *m*/*z* 43 is not the most prominent ion. This means that each ATX compound gives rises to a spectrum whose response factor is not comparable and this fact could lead consequences in the quantification since no ATX analogues standards are available and ATX standard was used to quantify the other analogues. In fact, the quantification of one compound using no proper standard can induce errors up to 200% [28]. In addition, the affinity of each analogue and the nAChR can be different, which affects the signal and therefore the amount obtained by FP could be also different [21]. It has been demonstrated that the planarity, H-bonding, size and steric configuration of the ATX side chain moiety plays an important role in the affinity of the ATX analogues for the nicotinic acetylcholine receptor ion channel sites [29]. Therefore, these new analogues could have higher affinity for the nAChR than the ATX.

## 3. Materials and Methods

### 3.1. Reagents and Materials

Pure ATX-a fumarate salt was purchased from Abcam^®^ (Abcam plc, Cambridge, UK). Phosphate-bufffered saline solution (PBS) composition in mM: 137 mM NaCl (Panreac, Barcelona, Spain), 8.2 mM Na_2_HPO_4_ (Panreac, Barcelona, Spain), 1.5 mM KH_2_PO_4_ from Merk (Darmstadt, Germany), 3.2 mMKCl (Panreac, Barcelona, Spain), pH adjusted to 7.3 adding NaOH (Panreac, Barcelona, Spain). Methanol and acetonitrile were purchased from Panreac (Barcelona, Spain). Black 96-well polystyrene microplates, MicrotiterMicrofluor^®^ 1 were from Thermo scientific, Hudson, NH, USA. Flat-bottom were used in all experiments.

### 3.2. Cyanobacterial Strains and Culture Conditions

Three cyanobacterial isolates were selected from the LEGE Culture Collection (CIIMAR, Porto, Portugal). Those include the anatoxin-a producing strain (ATX+) *Anabaena* sp. LEGE X-002 (=strain ANA 37) and the two non-producing strains (ATX-) *Anabaena* sp. LEGE 00233 and *Anabaena* sp. LEGE 00234. Each isolate was grown aseptically in 500 mL batch cultures in Z8 medium. Culture conditions were as follows: 25 °C, under a light/dark cycle of 14:10 h and a light intensity (*i.e.*, irradiance) of 30–40 μmol m^−2^ s^−1^. At the beginning of the stationary phase of growth (approximately 40 days), cells were harvested by filtration with a nylon-net of 10 μm mesh. The collected biomass was then rinsed with distilled water and freeze-dried.

### 3.3. Processing of ATX Sample

To extract the toxins and minimize matrix effects an extraction protocol was employed. Twenty milligrams of lyophilised biomass were used for each one three *Anabaena* spp. Green algae dried powder were weighted. The samples were then re-suspended in 4 mL of methanol (75%). Next, three cycles of 30 s of ultrasounds were done keeping the tube into ice. Once the algal cells were broken, the mixture was centrifuged at 3000 rpm for 10 min at 25 °C. The pellet obtained was re-suspended and extracted again twice with 4 mL of methanol (75%). The supernatants were combined, evaporated and re-suspended again in 170 µL.

### 3.4. Nicotinic Acetylcholine Receptor

*Torpedo marmorata* fish were obtained alive from the Station Biologique de Roscoff (Roscoff, France), and kept in artificial seawater for about a week in the aquarium of the CNRS animal house in Gif sur Yvette (Gif sur Yvette, France), until been used to prepare membranes from the electric tissue. *Torpedo* electrocyte membranes rich in α1_2_β1γδ nicotinic acetylcholine receptors (nAChRs) were purified in a cold room (4 °C) according to procedures previously described [30] with some modifications, as reported recently [23]. Membranes enriched in nAChR (membrane-nAChR) were re-suspended in 5 mM glycine and stored at −80 °C. 

The receptor was used in PBS (pH = 7) composed of 130 mM NaCl, 1.5 mM NaH_2_PO_4_, 8.5 mM Na_2_HPO_4_, 0.1% Tween-20 (*v*/*v*) and 0.1% BSA (*w*/*v*).

### 3.5. Fluorescence Polarization

A derivative of fluorescein, succinimidylesther of carboxyfluorescein (FAM), was employed as fluorescent molecule. Membrane-receptor labeling was performed using a kit purchased from emp Biotech: Fluoro protein 498 Spin Labeling and Purification Kit (Berlin, Germany) that includes all chemicals, tools, and dye reactive, needed for the labeling and purification processes. The membrane-nAChR was mixed with a sodium bicarbonate solution, then the dye was added and the mixture reacted for 1 h at room temperature and protected from light. In order to avoid unspecific interactions or unstable ester bond formation between dye and receptor, hydroxylamine was used as stop reagent. Finally, spin columns were used for rapid and efficient purification of the receptor-dye conjugate. With this kit, the dye reacts with an amine group of the protein and forms a covalent amide linkage. The membrane-nAChR-F conjugates have fluorescence-excitation and fluorescence-emission maxima at around 498 nm and 522 nm, respectively. The conjugate was stored at −80 °C protected from light until use 2.7 mg/mL of membrane-nAChR were labeled with the Fluoro Spin 498 protein Labeling Kit (emp Biotech GmbH, Berlin, Germany). The final membrane-nAChR-F conjugate, 90% of protein recovery, was dissolved in PBS. To select the correct dilution factor to perform the experiment and its volume, it is necessary to measure the fluorescence intensity of a dilution series of the protein solution obtained from the labeling reaction. Six different dilution factors, ranging from 1:100 to 1:7500 were tested in a final volume of 250 µL (data not shown). From this experiment, 1:1000 was chosen.With this dilution factor the final protein label concentration in each well was 4.8 ng/mL. This concentration remained constant in all experiments.

Changes in the fluorescence polarization (FP) of membrane-nAChR-F were measured by means of the Multi-Mode Microplate Reader, Synergy™ 4 from Biotek (Winooski, VT, USA). The instrument detection modes include fluorescence Intensity, FP, time-resolved fluorescence, luminescence, and UV-visible absorbance. Two types of fluorescence detection systems are available with Synergy 4, filter-based and monochromator-based. The instrument is equipped with dichroic mirrors and polarizing filters for FP. For measurement of FP and Fluorescence, excitation and emission wavelengths of 485/20 and 528/20 were used for membrane-nAChR-Fconjugates, respectively.

The polarization degree of the emitted light (measured in millipolarization units, mP) is calculated by the following equation:

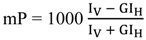
(1)
where *I*_V_ is the fluorescence intensity measured with vertical polarization excitation filters and vertical polarization emission filters (named parallel intensity), *I*_H_ is the fluorescence intensity measured with vertical polarization excitation filters and horizontal polarization emission filters (named perpendicular intensity), and *G* is a correction factor that accounts for the optical components of the instrument that affect the light beam depending on its polarization plane. 

The instrument was controlled using BioTek’s Gen5™ PC software (BioTek^®^ Instruments Inc, Winooski, Vermont, USA) for all measures, and data were exported to excel software for analysis. All results in this study are expressed as the mean ± SEM and the experiments were performed in triplicate with duplicate measurements per replicate. The results are expressed as fall of mP units (%) after toxin incubation.

### 3.6. LC-MS/MS Analysis

The LC-MS/MS analysis was performed by a combination of HPLC plus mass detector. The HPLC system, from Shimadzu (Kyoto, Japan), consists of two pumps (LC-10ADvp), autoinjector (SIL-10ADvp) with refrigerated rack, degasser (DGU-14A), column oven (CTO-10ACvp) and system controller (SCL-10Avp). This system is coupled to a QTRAP LC/MS/MS system from Applied Biosystems, (Bedford, MA, USA), which integrate a hybrid quadrupole-linear ion trap mass spectrometer equipped with an ESI source. The nitrogen generator is a Nitrocraft NCLC/MS from Air Liquide (Madrid, Spain).

The column used for cyanotoxins separations was a reverse phase C18 analytical column (100 mm × 4.6 mm i.d.) Chromatolith^®^ Performance RP-18efrom Merck, Darmstadt, Germany. The temperature was set at 40 °C. The composition of the mobile phase was: water (A) and acetonitrile (B), both containing 0.05% formic acid. Chromatographic separation was performed by gradient elution (23 min): starting with 2%–70% B for 12 min, then, 70% B was hold for 5 min and reducing afterwards to 2% B over 1 min and hold for 5 min until the next run. The mobile phase flow rate was 0.6 mL/min and the injection volume was 5 µL. Collision-induced dissociation (CID) in the ion-trap MS was performed. The electrospray ionization (ESI) source of QTRAP was operated with the following optimized values of source-dependent parameters: Curtain gas™ (Air Liquide, Madrid, Spain,): 20 psi, collision-activated dissociation gas (CAD): 6 psi, IonSpray Voltage: 4000 V, temperature: 450 °C, gas 1: 50 psi and gas 2: 50 psi. 

The mass spectrometer was operated in multiple reaction monitoring (MRM) detecting in positive mode analyzing the following transitions: anatoxin-a (ATX) (*m*/*z* 166 > 166, *m*/*z* 166 > 43), homoanatoxin-a (HATX) (*m*/*z* 180.1 > 163.1, *m*/*z* 180.1 > 145.1), dihidroanatoxin (H_2_ATX) (*m*/*z* 168.0 > 133.0, 168.0 > 150.0), dihidrohomoanatoxin (H_2_HATX) (*m*/*z* 182.0 > 147.0, 182.0 > 164.1), epoxyanatoxin-a (EpoxyATX-a) (*m*/*z* 182.0 > 164.1, 182.0 > 138.1) and epoxyhomoanatoxin-a (EpoxyhomoATX-a) (*m*/*z* 196.0 > 178.2, 196.0 > 138.1).

### 3.7. Data Analysis

All experiments were carried out at least three times using duplicates. Data were normalized and results were expressed as means ± SEM. Results were analyzed using the Student *t* test. A probability level of 0.05 or less was used for statistical significance.

## 4. Conclusions

In this paper, an effective and rapid functional method to detect ATXs in samples of *Anabaena* spp. is developed. The method is based on the change in FP when ATX-a binds to nAChR. By LC-MS/MS technique, the toxin profile of the *Anabaena* spp*.* culture was identified and besides ATX-a, chromatograms show three different ATX analogues, mass *m*/*z* 180.1. Since the quantity obtained by LC-MS/MS technique is considerably lower than those obtained by the FP assay, it seems that the analogues found in the *Anabaena* culture may have an important role in the toxicity of the sample. Thus, the existence of possible new cyanobacterial toxins like the ATX analogues shown in this study is a fact that should be taking into account for the safety and quality control of water and food products, in order to prevent health risks to consumers.
